# The Role of Answer Justification in Multiple-Choice Testing: Effects on Performance and Metacognitive Accuracy

**DOI:** 10.3390/bs15040477

**Published:** 2025-04-06

**Authors:** Spenser A. Clark, Michelle L. Rivers, Acacia L. Overono

**Affiliations:** 1 Department of Instructional Technology & Learning Sciences, Utah State University, Logan, UT 84322, USA; spenser.clark@usu.edu; 2 Department of Psychology, Santa Clara University, Santa Clara, CA 95053, USA; mrivers@scu.edu; 3 Psychology and Counseling Department, Utah Valley University, Orem, UT 84058, USA

**Keywords:** multiple-choice testing, assessment, metacognition, elaborative retrieval, self-explanation

## Abstract

Multiple-choice (MC) tests are widely used in educational settings but have been criticized for promoting passive recognition rather than active retrieval. Our research explores how adding a simple component to MC tests—answer justification—influences test performance and metacognitive accuracy. Across two experiments, university students studied a textbook chapter and completed either a standard MC test (MC-only group) or an MC test requiring them to justify their answers (answer justification group). Participants also provided predictive and postdictive metacognitive judgments. The results showed that the answer justification group significantly outperformed the MC-only group on an immediate test (Experiments 1 and 2) and scored numerically higher on a delayed test two days later (Experiment 2). Further, some initial evidence suggested that metacognitive accuracy was influenced by test type, but future research is needed. These findings support a retrieval-based explanation: generating answer justifications increases test performance by strengthening memory through elaborative retrieval. This study demonstrates that incorporating answer justification into MC tests may improve learning and metacognitive accuracy. We also offer practical suggestions for classroom implementation, considering that answer justification boosts test performance but also imposes a time cost compared to standard MC tests.

## 1. Introduction

What if answering a multiple-choice (MC) question became more than just a test of recognition? Imagine students engaging deeply with each question, weighing each response as a possible solution, and building stronger connections to the material to enhance their understanding. Standard MC questions, while efficient, often fail to achieve this level of cognitive engagement. How might we redesign MC tests to foster meaningful learning?

Our study explores how incorporating answer justification—a process where learners explain their chosen response—can enhance the educational value of MC questions. Drawing on principles of generative learning, we hypothesize that answer justification encourages elaborative retrieval and metacognitive monitoring, prompting learners to connect new information with prior knowledge and critically evaluate their reasoning. For the remainder of the Introduction, we discuss prior research that informs our specific predictions and outline how we evaluated these predictions across two experiments.

### 1.1. Improving Multiple-Choice Questions with Answer Justification

A significant challenge in education is to develop teaching and assessment methods that are efficient, adaptable, and effective in promoting meaningful learning. This challenge becomes even more pressing in settings like large classrooms and massive open online courses (MOOCs), where individualized feedback is limited. Multiple-choice (MC) questions are nearly ubiquitous in the classroom due to their ease of grading and scalability. However, they have been criticized for encouraging guesswork, relying on recognition rather than retrieval processes, and failing to measure complex understanding effectively (e.g., Brown & Abdulnabi, 2017; Greving & Richter, 2022; Ozuru et al., 2013). Despite these criticisms, recent research has shown that well-constructed MC tests can foster meaningful retrieval processes and improve subsequent recall of related information, challenging assumptions about their limitations (e.g., J. L. Little et al., 2012, 2019; J. L. Little & Bjork, 2015). For example, research by Sparck et al. (2016) demonstrated that a confidence-weighted MC format—in which test takers indicate their relative confidence among alternative responses—significantly enhanced later recall of relevant information compared to standard MC questions. This finding supports the idea that modifying MC tests to promote active, productive retrieval can offset their inherent reliance on passive recognition and ultimately improve learning outcomes. Another promising enhancement to standard MC questions is *answer justification*, which allows (e.g., Nield & Wintre, 1986) or requires (e.g., Bassett, 2016) learners to explain their reasoning for selecting a particular response at the time of testing.

Initial research on answer justification primarily examined its positive influence on student perceptions of MC questions (e.g., Gorrell & Cramond, 1988; Kottke, 2001; Nield & Wintre, 1986; Dodd & Leal, 1988). However, relatively few studies have explored its impact on student performance. Classroom-based research suggests that incorporating answer justification can enhance retrieval during testing compared to standard MC questions (Koretsky et al., 2016; McKeachie et al., 1955; Overono, in press; Papadopoulos et al., 2022). For example, Papadopoulos et al. (2022) gave students the option to complete in-class MC questions via an audience response system (e.g., “clickers”), with four of the eight questions including an optional justification component (limited to 140 characters). Students provided justifications for 76% of these questions, and the results showed that students earned high scores on questions that included the justification option compared to questions without a justification component. Additionally, similar studies that used MC and answer justification questions in classes (Koretsky et al., 2016; Overono, in press) found small to moderate effect sizes, suggesting an increased likelihood of selecting the correct answer when MC questions included a justification component. These classroom studies provide preliminary evidence that answer justification enhances performance on MC questions, but what explains its effectiveness?

### 1.2. The Role of Generative Learning in Answer Justification

Generative learning strategies, which involve actively constructing and interacting with new information, have been shown to enhance learning more effectively than passive study methods like rereading (for a review, see Fiorella & Mayer, 2016). Some of these techniques rely on creating connections between to-be learned material and prior knowledge (Martin & Pressley, 1991; Ozgungor & Guthrie, 2004), oneself (Symons & Johnson, 1997), or mnemonic devices (Putnam, 2015). A subset of generative learning strategies emphasize engagement through elaboration. One such technique, self-explanation (Chi et al., 1989), requires learners to generate explanations for themselves while learning new material. This process helps learners monitor understanding, identify knowledge gaps, and integrate novel information with prior knowledge, making it more meaningful (Bisra et al., 2018). Research indicates that generating explanations improves future test performance relative to study techniques that do not involve explanation-based elaboration (Tajika et al., 2007; Bisra et al., 2018).

Why is self-explanation effective? One account argues that the benefit of self-explanation on future test performance may be explained by elaborative encoding— by engaging in self-explanation, learners build a robust network of interconnected information, creating multiple retrieval pathways (M. A. McDaniel, 2023). This detailed network increases the likelihood of successful recall. Self-explanation and answer justification are similar in that they require individuals to explain various concepts, but unlike self-explanation, which typically occurs during the acquisition of new information, answer justification takes place at the time of testing. For this reason, its benefit on test performance may be better explained by the elaborative retrieval account. This account suggests that the act of justifying an answer strengthens retrieval by generating additional cues linked to the target memory (M. A. McDaniel, 2023); that is, when justifying their responses, learners may engage in retrieval around additional cues, increasing the chances of recalling the correct answer.

This retrieval-based explanation aligns with the proposed explanation for why MC questions with competitive alternatives—plausible but incorrect responses—lead to increased performance on later tests compared to questions that use noncompetitive alternatives (J. L. Little et al., 2012; J. L. Little & Bjork, 2015). For example, J. L. Little et al. (2019) examined whether participants given well-crafted MC questions spontaneously recall relevant details related to incorrect alternative responses. Participants were given MC trivia questions and prompted to either explain why each alternative response was incorrect (Experiment 1) or justify their chosen answer (Experiment 2). The results showed that participants did spontaneously retrieve information related to incorrect alternatives, and notably those retrievals were associated with increased performance on later cued-recall tests related to those alternatives. If participants are spontaneously retrieving information at test, requiring answer justification may further encourage this process and improve performance both at test and in subsequent tests.

In answer justification, rather than relying on competitive alternatives to encourage deeper thinking, the act of justification itself prompts elaborative retrieval. To understand, consider the MC question: “Which of these would belong in a mineral collection?” with the answer choices *granite*, *basalt*, *quartz*, and *schist*. A student answering traditionally might simply select the most recognizable option, focus only on the first plausible choice, and stop once they feel confident in their selection. However, when required to justify their answer, the student must evaluate each option more thoroughly, potentially retrieving information about minerals, as well as metamorphic and igneous rocks. This elaborate retrieval process increases the likelihood of correctly identifying quartz as the only true mineral in the list and may even strengthen future recall.

Additionally, answer justification may promote effortful retrieval in ways that standard MC questions do not (Greving & Richter, 2022). While elaborative retrieval strengthens memory by increasing the number of retrieval routes to a target memory, MC questions can often be answered through recognition or chance (Greving & Richter, 2022). Unless specifically designed to circumvent this (J. L. Little & Bjork, 2015), learners may not feel motivated to invest significant cognitive effort. In contrast, questions that require justification demand additional processing and provide some structure and accountability, making it more likely that learners engage in effortful retrieval and generate elaborative information, ultimately enhancing learning outcomes.

### 1.3. The Role of Metacognition in Answer Justification

Metacognition—the process of evaluating and regulating one’s understanding and cognitive performance (Flavell, 1979)—may contribute to improved test performance when MC questions include answer justifications. Effective metacognitive monitoring involves assessing how well information is understood and can directly inform what decisions learners make about their learning process (i.e., metacognitive control, e.g., Dunlosky & Rawson, 2012). Metacognitive monitoring is often measured by having learners make judgments about their learning at various points and comparing these judgments to objective indicators of performance (Nelson & Narens, 1990; Schraw, 2009). For instance, a learner might study material, estimate their likelihood of remembering it on a 0–100 scale, and then take a test. The alignment between their judgment and test performance reflects their metacognitive monitoring accuracy—how well they calibrate confidence to actual performance. These judgments can be made before an assessment (predictions) or after (postdictions) and at varying levels of specificity, such as across an entire test (global judgments) or within specific topics (topic-level judgments).

How might answer justification influence metacognitive accuracy? According to the *cue-utilization framework* (Koriat, 1997), metacognitive judgments are not based on direct access to memory strength but rather on inferences drawn from various cues. By requiring learners to retrieve and articulate their reasoning, answer justification may direct attention to a wider array of meaningful cues about their memory and understanding that are more diagnostic of test performance, rather than superficial cues like familiarity with the learning material (T. D. Griffin et al., 2019a). For example, research has shown that engaging in self-explanation while reading text material improves metacognitive accuracy by helping learners recognize gaps in their knowledge (e.g., T. D. Griffin et al., 2008; Fukaya, 2013). In the context of MC questions, answer justification likely operates similarly—encouraging learners to critically evaluate their responses could lead to a better alignment between perceived and actual comprehension. Furthermore, when people anticipate having to explain their reasoning, they may be more likely to base their predictions on relevant cues, leading to more accurate judgments. For example, research suggests that students tend to align their metacognitive judgments with the type of test they expect, indicating that expectations can shape the way that students evaluate their understanding (T. D. Griffin et al., 2019b; Thiede et al., 2011; Wiley et al., 2008). Thus, MC questions with answer justification may lead to more accurate predictive and postdictive judgments relative to standard MC questions.

### 1.4. Present Research

Answer justification may offer educators a simple yet effective way to enhance students’ recall during tests by encouraging them to self-generate cues that promote elaborative retrieval. Additionally, it may improve metacognitive accuracy, helping students better assess their own understanding. To examine these potential benefits, we analyzed differences in test performance and metacognitive judgments between participants who were required to provide answer justifications for MC questions and those who were not.

Experiment 1 investigated these ideas by having participants study an introductory geology text before being randomly assigned to one of two groups. Both groups provided global and topic-level metacognitive judgments before and after the test, which consisted of 12 MC questions. However, only the experimental group was required to provide answer justifications. We hypothesized that participants who justified their answers would score higher on the test and that those expecting to justify their answers would demonstrate higher metacognitive accuracy (for both global and topic-level judgments) both before and after testing compared those who answered MC questions without justification.

In Experiment 2, we attempted to replicate the findings of Experiment 1 and examine how answer justification influences subsequent retrieval on a delayed MC test compared to MC testing alone. Similarly, we hypothesized that participants in the answer justification group would outperform participants in the MC-only group on both the immediate and delayed test.

## 2. Experiment 1

### 2.1. Methods

#### 2.1.1. Sample

Participants were 110 college students from an open enrollment university in the Intermountain West of the United States. Participants were recruited from a departmental participant pool consisting of undergraduate students who are required to participate in research for course credit. The majority of participants were from Introductory Psychology courses. Participants were excluded if they had inconsistencies in providing metacognitive judgments for either global or topic-level judgments (for example, giving a 0% for a global judgment and then values for topic-level judgments or vice-versa, *n* = 13) or if they admitted to cheating by using outside resources during the test (*n* = 18). After applying these exclusion criteria, the final sample consisted of 79 participants. Participants were randomly assigned to one of two groups: the multiple-choice-only (MC-only) group (*n* = 40) or the answer justification group (*n* = 39). To our knowledge, Experiment 1 was the first experiment of its kind to investigate the influence of answer justification on multiple-choice test performance in a non-classroom setting with geology materials. We attempted to reach approximately 40 participants per group, similar to the group sizes of Papadopoulos et al. (2022) with group sizes of 54 and 44.

#### 2.1.2. Materials

The study was conducted using an online survey administered through Qualtrics. All participants provided informed consent.

#### 2.1.3. Procedure

**Figure 1:**
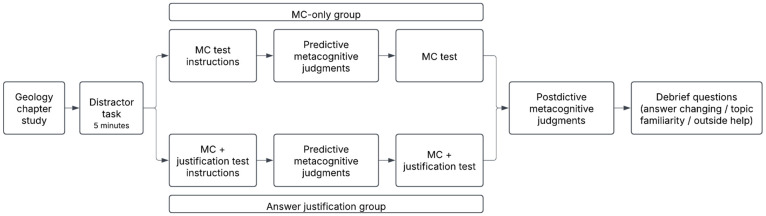
Procedure for Experiment 1.

The full experimental procedure is depicted in Figure 1.

**Learning Phase.** Both groups read a chapter from an introductory geology textbook covering fundamental geology concepts (Earle, 2019). The chapter, approximately 3150 words long, included images to illustrate key concepts. Participants were allowed to read at their own pace. Upon completing the reading, they engaged in a five-minute distractor task, which involved playing an online fishing game.

After the distractor task, participants were informed about the type of test they would take. Those in the MC-only group were told they would complete a standard MC test, whereas those in the answer justification group were instructed that, in addition to answering MC questions, they would need to “explain why they selected” each response.

**Predictions.** Following these test-specific instructions, participants were asked to estimate their expected test performance. Firstly, they provided an estimate of the percentage of questions (0–100%) they believed they would answer correctly overall (global judgment). After, participants were given four topics for each content subsection of the geology text (*defining geology*, *minerals and rocks*, *plate tectonics*, and *geological time*) and were again asked to estimate the percentage of questions for each topic they would answer correctly (topic-level judgments). Each topic corresponded to a different number of questions, ranging from 2 to 5 questions. The different numbers of questions reflected the varying length of each subsection of the text.

**Figure 2:**
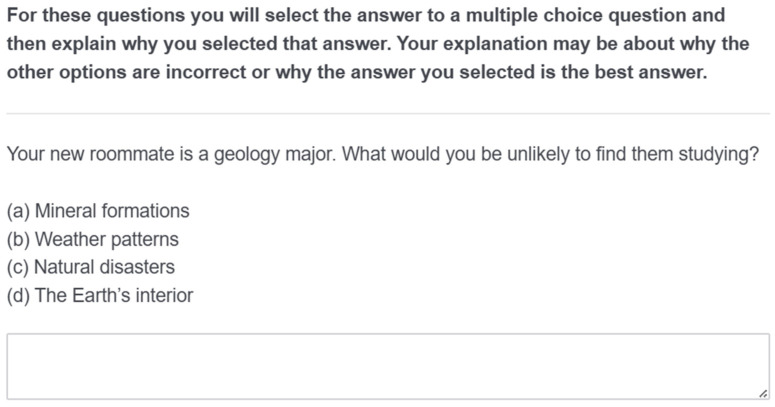
Example of the answer justification test format.

**Test.** The test consisted of 12 MC questions covering the material from the textbook chapter participants read. Participants in the MC-only group answered each question in a standard multiple-choice format. All questions were presented on a single page, and participants had unlimited time to respond and modify their answers as needed. Participants in the answer justification group received the same question stems and response choices but were also required to provide a brief explanation for each response in a text entry box (Figure 2). The survey was designed to ensure that all participants answered every MC question and, for those in the answer justification group, entered text in the justification box before proceeding. Test questions were presented in a fixed order for all participants.

**Postdictions and Debrief.** After completing the test, participants in both groups provided postdictive global and topic-level judgments, using the same format as their predictions. Before concluding the experiment, participants were asked, “Did you use anything aside from your own memory to answer the questions about geology (for example google, a friend, notes, etc.)?” Participants who answered “yes” to this question were excluded from analyses. Participants were also asked about their familiarity with geology concepts presented in the chapter (on a 5-point scale ranging from “not familiar at all” to “extremely familiar”) and if they were in the answer justification group, how frequently they recalled changing their answers (on a 5-point scale ranging from “never” to “always”).

### 2.2. Results

#### 2.2.1. Test Performance

**Figure 3:**
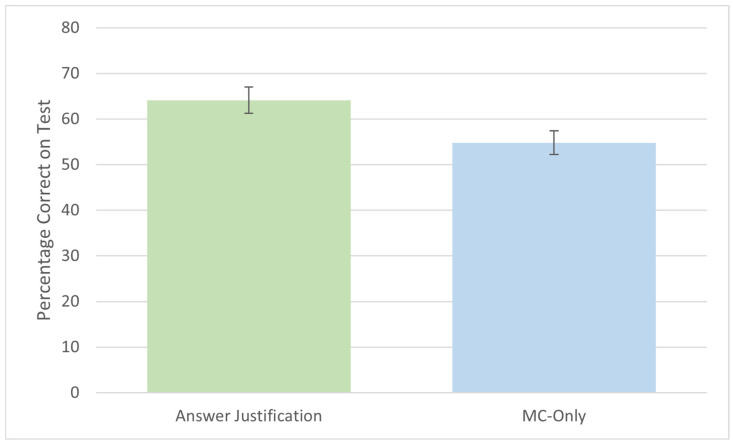
Performance on test for MC-only and answer justification groups in Experiment 1. Error bars depict the standard error of the mean.

Test performance was measured as the average percentage of correct responses across all 12 MC questions within each group and is presented in Figure 3. For the answer justification group, only the accuracy of the MC responses was considered; written justifications were not evaluated for correctness. The results indicated a significant difference in performance between groups, with the answer justification group scoring higher (*M* = 64%, *SD* = 18) than the MC-only group (*M* = 55%, *SD* = 16), *t*(77) = 2.39, *p* = 0.019, *d* = 0.54.

#### 2.2.2. Metacognitive Accuracy

Descriptive statistics for all performance and judgment variables are presented in Table 1. We compared judgment accuracy for predictive and postdictive judgments by group. To measure metacognitive accuracy, we computed bias scores (Schraw, 2009). Bias is the difference between one’s judgment and actual performance on a given topic, and it can reveal whether participants are overconfident (indicated by positive bias scores) or underconfident (indicated by negative scores) about their performance.

**Global Judgments.** We calculated bias by subtracting participants’ overall performance on the multiple-choice test from their global judgments. A 2 (judgment type: predictive, postdictive) × 2 (group: MC-only, answer justification) mixed ANOVA on bias scores revealed no significant main effect of bias type, *F*(1, 77) = 3.12, *p* = 0.081, η_p_^2^ = 0.04, or group, *F*(1, 77) = 3.35, *p* = 0.071, η_p_^2^ = 0.04. The interaction between bias type and group was significant, *F*(1, 77) = 5.73, *p* = 0.019, η_p_^2^ = 0.07.

Follow-up paired-samples *t*-tests revealed that this interaction was driven by a significant difference in bias scores between predictive and postdictive bias for the MC-only group, *t*(39) = 2.77, *p* = 0.009, *d* = 0.44, such that predictive bias (*M* = 9, *SD* = 21) was higher than postdictive bias (*M* = −1, *SD* = 24). However, for the answer justification group, there was no significant difference between predictive bias (*M* = −5, *SD* = 22) and postdictive bias (*M* = −3, *SD* = 23), *t*(38) = 0.48, *p* = 0.635, *d* = 0.08. Thus, participants in the MC-only group improved their bias scores from predictions to postdictions, whereas participants in the answer justification group had low bias scores at the time of predictions.

**Topic-Level Judgments.** We computed bias by subtracting participants’ actual topic performance from their topic-level judgments. We then computed an average across topics.

A 2 (judgment type: predictive, postdictive) × 2 (group: MC-only, answer justification) mixed ANOVA on topic-level bias scores revealed no significant main effect of judgment type, *F*(1, 77) = 2.23, *p* = 0.139, η_p_^2^ = 0.03, or group, *F*(1, 77) = 1.49, *p* = 0.188, η_p_^2^ = 0.02. The interaction between judgment type and group was significant, *F*(1, 77) = 4.53, *p* = 0.037, η_p_^2^ = 0.06.

Follow-up paired-samples *t*-tests revealed that this interaction was driven by a significant difference between predictive and postdictive bias for the MC-only group, *t*(39) = 2.33, *p* = 0.025, *d* = 0.37, such that predictive bias (*M* = 6, *SD* = 24) was higher than postdictive bias (*M* = −2, *SD* = 28). However, for the answer justification group, there was no significant difference between predictive bias (*M* = −5, *SD* = 23) and postdictive bias (*M* = −4, *SD* = 23), *t*(38) = −0.51, *p* = 0.614, *d* = 0.08; that is, participants in the answer justification group were already fairly accurate (and even slightly underconfident, on average) in their topic-level judgments prior to taking the multiple-choice test.

#### 2.2.3. Familiarity Ratings and Self-Reported Answer Changing

Overall, participants reported moderate levels of familiarity with the content. Participants in the answer justification group reported numerically higher ratings of content familiarity (*M* = 2.44, *SD* = 0.79) than the MC-only group (*M* = 2.17, *SD* = 0.90), but this difference was not statistically significant, *t*(77) = 1.37, *p* = 0.176, *d* = 0.31. Participants in the answer justification group were also asked to report how frequently they changed their answers on the test and the majority of participants indicated they changed their answers “sometimes” (see the top of Table 2).

### 2.3. Discussion

The results of Experiment 1 highlight the effectiveness of answer justification in promoting accurate retrieval during testing. Participants who provided justifications selected more correct responses on MC questions compared to those who answered standard MC questions without justification.

Additionally, patterns in metacognitive judgment accuracy differed between groups. The MC-only group overestimated their performance before the test but adjusted their postdictions, improving their accuracy. In contrast, the answer justification group showed no significant change in metacognitive accuracy from predictions to postdictions, suggesting that the expectation to justify answers may have led to more realistic performance predictions from the outset. However, our measures of metacognitive accuracy are limited, a point we return to in the General Discussion.

Experiment 1 had several limitations that we aimed to address in Experiment 2. Firstly, we were slightly under-powered to detect the observed effect of answer justification on test performance, so in Experiment 2, we attempted a high-powered replication of this outcome. Secondly, the percentage-based metacognitive judgments may have confused participants and led to some errors in providing judgments. Thus, in Experiment 2, we had participants make their judgments on the same scale as performance to reduce ambiguity in interpreting our bias scores (as recommended by Dunlosky et al., 2016). Further, in Experiment 1, we did not measure time on the test, which could offer insights into the relative efficiency of each test type. This measurement was added in Experiment 2.

## 3. Experiment 2

The results of Experiment 1, along with previous research, suggest that answer justification improves performance on MC tests compared to standard MC questions. If the elaborative retrieval account is correct and answer justification engages elaborative retrieval processing during testing, it should also improve performance on subsequent tests, functioning as a form of test-potentiated learning or retrieval practice (S. K. Carpenter et al., 2022; Roediger & Butler, 2011). Retrieval practice, a learning strategy that involves actively recalling information from memory, has been shown to enhance long-term retention compared to restudy (Rowland, 2014). More recently, work has begun to explore how retrieval practice, combined with forms of elaborative encoding, may increase learning even more than retrieval practice alone (for a review, see M. A. McDaniel, 2023). While past research demonstrates that MC questions can be effective for retrieval practice (e.g., J. L. Little et al., 2012; Smith & Karpicke, 2014), their benefits depend on question construction. If answer justification encourages elaboration similar to how MC questions with competitive alternatives function (e.g., J. L. Little et al., 2012), then answer justification should also enhance delayed test performance compared to MC-only tests, making it a more effective long-term strategy than standard MC testing.

To investigate this possibility, Experiment 2 sought to replicate the findings of Experiment 1 and assess whether answer justification also functions as an effective encoding strategy, improving retrieval of the same information after a longer delay. Participants underwent the same procedure as in Experiment 1 and then returned for a second experimental session two days later, during which they took a standard multiple-choice test on the same content.

### 3.1. Methods

#### 3.1.1. Sample

Experiment 2 included 257 undergraduate students from the same university participant pool as Experiment 1. Prior to data collection, a sample size analysis was conducted using G*Power 3.1.9.7 (Faul et al., 2009) for an independent sample *t*-test, assuming 80% power and a medium effect size (*d* = 0.5, similar to the effect of answer justification on performance observed in Experiment 1), which suggested a sample size of 64 per group, or 128 participants in total. To ensure adequate power, we continued data collection until 132 participants had completed the second session, at which point we began our analysis.

Participants were excluded if they provided impossible metacognitive judgments (for example, indicating they would answer four questions correctly on a topic with only three corresponding questions) in providing either global or topic-level judgments (*n* = 19), admitted to cheating (*n* = 13), or failed to complete the full procedure in Session 1 (*n* = 2). Due to the multi-day nature of the study, we experienced a significant dropout rate (53%) between the first and second sessions, resulting in a final sample size of 238 participants for Session 1 (*n* = 126 MC-only, 112 answer justification) and 112 participants for Session 2 (*n* = 57 MC-only, 55 answer justification).

#### 3.1.2. Materials

The textbook chapter, test questions, and explanations of MC questions and answer justification were identical to Experiment 1. We modified the prediction and postdiction format. Instead of estimating the percentage of correct answers, participants were asked to predict the number of questions they expected to answer correctly (e.g., “*The test you are about to take consists of 12 questions. How many questions do you think you will answer correctly?*”). This adjustment aimed to make participants’ self-assessments clearer and more consistent.

#### 3.1.3. Procedure

Experiment 2 consisted of two online sessions. The first session followed the same procedure as Experiment 1, including the learning phase, immediate test phase, and the predictive and postdictive judgments. The second session opened 48 h later, and participants had 12 h to complete the session. During this session, all participants completed a standard MC test. Participants also completed a final (postdictive) judgment at this time, but due to an unfortunate coding error, these data were uninterpretable and are thus not reported here. At the end of the second session, participants were asked the same questions as in Experiment 1 regarding the use of additional resources (e.g., Google, notes, friends) to ensure test integrity. Participants were also timed during the immediate test to examine how long MC-only and answer justification groups took to answer their respective question types (and provide a measure of learning efficiency).

### 3.2. Results

#### 3.2.1. Test Performance

We first analyzed test performance across both the immediate and delayed tests using a 2 (group: MC-only, answer justification) × 2 (test timing: immediate, delayed) mixed ANOVA. The results showed a main effect of group, *F*(1, 110) = 4.70, *p* = 0.032, η_p_^2^ = 0.04, with the answer justification group (*M* = 67%, *SE* = 2) outperforming the MC-only group (*M* = 63%, *SE* = 2). There was no main effect of test timing, *F*(1,110) = 1.86, *p* = 0.175, η_p_^2^ = 0.02, and no interaction, *F*(1,110) = 3.50, *p* = 0.064, η_p_^2^ = 0.03. These results suggest that the benefits of answer justification persist over a delay.

We next examined participants’ performance in Session 1, which was analyzed using an independent-samples *t*-test. Consistent with Experiment 1, participants in the answer justification group (*M* = 67%, *SD* = 18) outperformed those in the MC-only group (*M* = 62%, *SD* = 17), *t*(236) = 2.38, *p* = 0.018, *d* = 0.31. These results successfully replicate the positive effect of answer justification on test performance.

We then analyzed group differences for the 47% of participants who completed the 48 h delayed MC test. Numerically, participants in the answer justification group (*M* = 67%, *SD* = 17) scored higher than those in the MC-only group (*M* = 61%, *SD* = 17), but the results were only marginally significant *t*(110) = 1.82, *p* = 0.071, *d* = 0.34.

#### 3.2.2. Time on Test

We examined the length of time each group spent on the immediate test. Participants in the answer justification condition took significantly more time to complete the test (*M* = 14.19 min, *SD* = 10.52) than participants in the MC-only group (*M* = 3.98, *SD* = 3.05), *t*(236) = 10.4, *p* < 0.001, *d* = 1.35. For each participant, we calculated *gains per minute* (GPM; see Rawson & Dunlosky, 2013) by dividing their test performance by the average number of minutes spent during the test. On average, participants in the MC-only group had a GPM of 0.20 (*SD* = 0.11), while the answer justification group was 0.06 (*SD* = 0.03). These results indicate that while answer justification increases overall performance, it takes substantially longer for participants to complete.

#### 3.2.3. Metacognitive Accuracy

Descriptive statistics for all performance and judgment variables are presented in Table 3. As a reminder, judgments were made for the corresponding number of questions (e.g., for each topic) rather than on a percentage scale.

**Global Judgments.** As in Experiment 1, we computed bias by subtracting participants’ performance from their judgments. A 2 (judgment type: predictive, postdictive) × 2 (group: MC-only, answer justification) mixed ANOVA on bias scores revealed a significant main effect of judgment type, *F*(1, 236) = 18.12, *p* < 0.001, η_p_^2^ = 0.07, with predictive bias (*M* = −0.13, *SE* = 0.17) being lower than postdictive bias (*M* = −0.71, *SE* = 0.16). The main effect of group was not significant, *F*(1, 236) = 0.93, *p* = 0.335, η_p_^2^ = 0.004. The interaction was significant, *F*(1, 236) = 7.60, *p* = 0.006, η_p_^2^ = 0.03.

Follow-up paired-samples *t*-tests revealed that this interaction was driven by a significant difference in bias scores between predictive and postdictive bias for the MC-only group, *t*(125) = 5.68, *p* < 0.001, *d* = 0.51, such that predictive bias (*M* = 0.21, *SD* = 2.41) was higher than postdictive bias (*M* = −0.75, *SD* = 2.25). However, for the answer justification group, there was no significant difference between predictive bias (*M* = −0.46, *SD* = 2.71) and postdictive bias (*M* = −0.66, *SD* = 2.64), *t*(111) = 0.94, *p* = 0.335, *d* = 0.09.

**Topic-Level Judgments.** A 2 (judgment type: predictive, postdictive) × 2 (group: MC-only, answer justification) mixed ANOVA on bias scores revealed a significant main effect of judgment type, *F*(1, 236) = 22.37, *p* = < 0.001, η_p_^2^ = 0.09, with predictions showing a very small degree of overconfidence (*M* = 0.003, *SE* = 0.04) and postdictions showing underconfidence (*M* = −0.16, *SE* = 0.04). The main effect of group was not significant, *F*(1, 236) = 0.69, *p* = 0.408, η_p_^2^ = 0.003. The interaction between judgment type and group was significant, *F*(1, 236) = 5.93, *p* = 0.016, η_p_^2^ = 0.03.

Follow-up paired-samples *t*-tests revealed that this interaction was driven by a significant difference between predictive and postdictive bias for the MC-only group, *t*(125) = 5.82, *p* < 0.001, *d* = 0.52, such that predictive bias (*M* = 0.08, *SD* = 0.57) was higher than postdictive bias (*M* = −0.17, *SD* = 0.57). However, for the answer justification group, there was no significant difference between predictive bias (*M* = −0.07, *SD* = 0.70) and postdictive bias (*M* = −0.15, *SD* = 0.70), *t*(111) = 1.43, *p* = 0.164, *d* = 0.14.

#### 3.2.4. Familiarity Ratings and Self-Reported Answer Changing

Familiarity ratings did not differ for participants in the answer justification group (*M* = 2.09, *SD* = 0.89) or the MC-only group (*M* = 2.18, *SD* = 0.90), *t*(110) = 0.61, *p* = 0.542, *d* = 0.12. Similar to Experiment 1, participants in the answer justification group most frequently stated that they changed their answers “sometimes” (see the bottom of Table 2).

### 3.3. Discussion

The results of Experiment 2 successfully replicate the findings from Experiment 1, demonstrating that participants in the answer justification group outperformed those in the MC group on the immediate test. Additionally, the results reaffirmed that the predictive and postdictive bias scores varied depending on the testing group. Performance on the standard MC test administered 48 h later showed a numerical advantage for the answer justification group over MC-only, but analyses did not reach significance.

## 4. General Discussion

**Figure 4:**
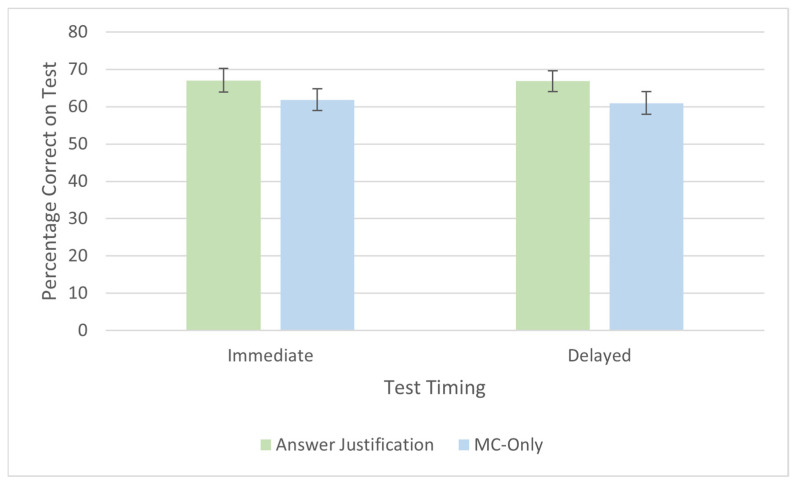
Performance on immediate test and delayed test in Experiment 2. Error bars depict the standard error of the mean.

Across two experiments, we demonstrated that answer justification is an effective technique for enhancing test performance compared to standard MC questions. In our first experiment, participants who provided justifications were more likely to select the correct response than those who simply answered the questions alone (Figure 3). Our second experiment replicated this finding and explored how answer justification influenced learning after a delay (Figure 4). In the delayed test (48 h later), we found the same numerical effect in the same direction as the immediate test, where participants in the answer justification group scored higher than those who did not have the option to justify their answers, but these results were nonsignificant. Overall, our three comparisons of answer justification to MC-only tests on MC test performance yielded small to medium effect sizes (Experiment 1: *d* = 0.54; Experiment 2, immediate yest: *d* = 0.31, delayed test: *d* = 0.34). These results suggest that answer justification may involve elaborative retrieval, but further exploration is needed.

### 4.1. Metacognitive Implications

Our findings provide some initial evidence that anticipating an answer justification task may influence metacognitive judgments (see Table 1 and Table 3). Participants in the MC-only group exhibited a significant shift in their predictive and postdictive accuracy (in both global and topic-level judgments), initially overestimating their performance and becoming more accurate (or even underconfident) in their postdictions. This outcome is consistent with previous research showing a *postdiction superiority effect*, likely driven by retrieval-based processes that occur during a test (e.g., remembering what happened when questions were answered; Pierce & Smith, 2001; Rivers et al., 2019). In contrast, participants in the answer justification group showed more stable metacognitive judgments. Interestingly, participants who anticipated and completed answer justifications tended to exhibit underconfidence in both global and topic-level judgments.

However, because our measure of metacognitive accuracy (bias) is a derived measure that includes both judgment magnitude and test performance, our metacognitive outcomes should be interpreted with caution; that is, we cannot conclude that differences in bias scores are due to better monitoring ability for the answer justification group (Dunlosky et al., 2016). Thus, further systematic investigation will be needed to evaluate the influence of answer justification on metacognitive monitoring ability. Specifically, future research should have learners make metacognitive judgments for each multiple-choice question (with or without answer justification), which would allow for a cleaner measure of bias and a calculation of relative metacognitive accuracy (i.e., how well learners can discriminate between material they know well versus not as well).

### 4.2. Alternative Explanations

While our findings provide some support for a retrieval-based explanation, alternative explanations for the benefits of answer justification should be considered. Some researchers suggest that answer justification may reduce test anxiety, as students often prefer question formats that allow them to explain their reasoning rather than simply selecting an answer (Nield & Wintre, 1986). One potential source of anxiety in MC questions is a mismatch between a student’s knowledge and the given answer choices. Providing a justification option may give students a greater sense of control, reducing test-related stress (McKeachie et al., 1955). In support of this idea, students have expressed preference for question formats that allow them to more fully articulate what they have learned—such as essay and short-response—even while acknowledging that MC questions are “easier” (Nield & Wintre, 1986). However, test anxiety alone is unlikely to explain our results. Unlike prior studies conducted in high-stakes classroom settings, our research took place in a low-stakes laboratory environment, where anxiety was presumably lower. Moreover, it is possible that answer justification increased effort rather than reduced stress.

Another possible explanation is that better performance on the delayed test in Experiment 2 may be attributed to retrieval success rather than elaborative retrieval. Previous work on the retrieval practice has demonstrated that success during retrieval practice strongly predicts later test performance (Pan & Rickard, 2018; Rowland, 2014, though see Chan et al., 2024 for another perspective). In our study, participants in the answer justification group for Experiment 2 may have performed better on the delayed test simply because they answered more questions correctly on the immediate test, rather than due to more elaborative retrieval related to items brought to mind during justification. Examining the results of Experiment 2, participants in both the answer justification group and MC-only group did show a strong, positive correlation between performance at immediate and delayed tests (*r* = 0.80, *p* < 0.001, *r* = 0.77, *p* < 0.001, respectively). These correlations suggest that retrieval success had some relationship with later performance, though such a relationship was similar across both groups and may not explain the entire benefit of answer justification.

### 4.3. Limitations and Future Directions

Several methodological limitations should be acknowledged. Firstly, data collection was conducted entirely online via Qualtrics, which limited our ability to control or monitor participant behavior. Although participants were asked to self-report cheating, it is possible that some undisclosed cheating occurred, affecting the outcomes. Secondly, there was a substantial dropout rate in Experiment 2 (53% of participants did not complete the delayed test), which reduced our sample size for the second session and limited the generalizability of our results. Further, our metacognitive questions changed from Experiment 1 to Experiment 2. We made this change to address errors in participant responding, but this change in measurement across the two studies makes them challenging to compare.

Future work could investigate other applications of answer justification as well as its limitations. One avenue of research could explore how using answer justification as an encoding technique influences performance on delayed *transfer* tests. Much like J. L. Little et al. (2019), if engaging in justification causes participants to spontaneously retrieve details surrounding the question or related responses, that elaborative retrieval could lead to enhanced performance on related transfer questions at a later test.

Another important direction for future research is examining individual differences to determine how they influence the effectiveness of answer justification. In our experiments, participants reported moderate levels of familiarity with the geology content. However, self-reported familiarity is limited, and an important direction for future research would be to explore the interaction between answer justification and prior knowledge (as it plays a role in other learning techniques, e.g., Witherby & Carpenter, 2022). Understanding how different learner characteristics affect the benefits of justification could help tailor instructional strategies to diverse student populations. Additionally, research should compare answer justification to other generative learning strategies, such as self-explanation (Renkl & Eitel, 2019), elaborative interrogation (e.g., M. A. McDaniel & Donnelly, 1996), and retrieval practice with open-ended responses, to better understand its relative effectiveness and underlying mechanisms. Further comparisons could also be made with social learning strategies like *peer instruction*, in which students first answer multiple-choice questions individually and then discuss their responses with peers before re-answering (Crouch & Mazur, 2001; Tullis & Goldstone, 2020). This comparison could help determine whether collaborative reasoning provides similar or additional benefits compared to individual justification.

Finally, future studies should examine classroom implementation factors to better understand how answer justification functions in real-world educational settings, including high-stakes exams, online learning platforms, and adaptive testing environments. Understanding how factors like time constraints, instructional support, and grading considerations influence its effectiveness could help educators integrate this technique more effectively. By addressing these questions, future research can refine our understanding of when and how answer justification is most beneficial for learning and assessment.

## 5. Conclusions

Answer justification is a simple addition to MC questions that can be easily adopted by instructors. Our results indicate that adding answer justification enhances performance during testing. Unlike other best practices for MC tests, which emphasize time spent developing questions with appropriate competitive responses, answer justification may be adopted for a wide variety of question types to introduce an added element of difficulty and retrieval.

While answer justification offers clear benefits, its adoption in classroom settings requires careful planning. One consideration is the increased time required for completion, as answer justification takes longer than standard multiple-choice questions. In Experiment 2, we found that participants spent significantly more time on answer justification questions than MC-only questions (about 10 more minutes on average). Instructors may need to adjust test length or allocate additional time for justifications. Another factor is question suitability; answer justification probably works best for conceptual and application-based questions rather than simple definition-based recall. For example, a question like “What is a mineral?” may not be as effective in this format as “Which of the following is a mineral?” because the latter allows for a more meaningful explanation.

Finally, in classroom settings, answer justifications provide further insight into student thinking compared to standard MC. Students may spontaneously reveal misconceptions, gaps in their thinking, or admit lack of knowledge in their responses. These rich responses provide an opportunity for individualized interventions to address misunderstandings.

## Figures and Tables

**Table 1:** Average magnitude (percentage) of participants’ global and topic-level metacognitive judgments and performance in Experiment 1.

		Topic-Level Judgments			
	Global Judgments	Defining Geology	Minerals and Rocks	Plate Tectonics	Geologic Time
	MC-Only Group				
Predictive Judgments	64 (3)	77 (4)	62 (4)	56 (4)	45 (4)
Performance	55 (16)	68 (5)	47 (5)	63 (3)	35 (5)
Postdictive Judgments	54 (4)	62 (5)	52 (5)	50 (5)	50 (4)
	Answer Justification Group				
Predictive Judgments	59 (4)	72 (4)	60 (4)	53 (4)	52 (4)
Performance	64 (18)	77 (5)	57 (5)	72 (3)	42 (5)
Postdictive Judgments	61 (4)	71 (5)	60 (5)	55 (4)	56 (4)

Note: Standard errors are in parentheses.

**Table 2:** How often participants in the answer justification group reported changing their responses to multiple-choice questions as they were writing explanations in Experiments 1 and 2.

	Never	Sometimes	About Half the Time	Most of the Time	Always
Experiment 1	28%	54%	10%	8%	0%
Experiment 2	24%	53%	16%	6%	2%

**Table 3:** Average magnitude of participants’ global and topic-level metacognitive judgments and performance in Experiment 2.

		Topic-Level Judgments			
	Global Judgments	Defining Geology	Minerals and Rocks	Plate Tectonics	Geologic Time
	MC-Only Group				
Predictive Judgments	7.60 (0.19)	1.79 (0.04)	2.02 (0.06)	2.66 (0.10)	1.23 (0.06)
Immediate Test Performance	7.40 (0.18)	1.60 (0.05)	1.61 (0.08)	3.25 (0.10)	0.94 (0.07)
Postdictive Judgments	6.64 (0.20)	1.70 (0.04)	1.69 (0.07)	2.27 (0.11)	1.06 (0.06)
	Answer Justification Group				
Predictive Judgments	7.59 (0.20)	1.83 (0.04)	1.98 (0.07)	2.66 (0.10)	1.29 (0.06)
Immediate Test Performance	8.04 (0.20)	1.70 (0.05)	1.87 (0.08)	3.56 (0.11)	0.92 (0.07)
Postdictive Judgments	7.38 (0.23)	1.80 (0.06)	1.96 (0.08)	2.59 (0.12)	1.10 (0.06)

Note: Standard errors are in parentheses. The immediate test and global judgments were out of 12 questions; topics varied in the number of questions (defining geology: 2 questions, minerals and rocks: 3 questions, plate tectonics: 5 questions, geologic time: 2 questions).

## Data Availability

The data presented in this study are openly available in the Open Science Framework (OSF) at https://osf.io/x9hmv/?view_only=e2b80cfff261469a9b531146f815ef37 (accessed on 10 February 2025).

## Abbreviations

The following abbreviations are used in this manuscript:

MOOC	Massive open online courses
GPM	Gains per minute
MC	Multiple-choice
